# Feasibility of photoacoustic-guided teleoperated hysterectomies

**DOI:** 10.1117/1.JMI.5.2.021213

**Published:** 2018-02-08

**Authors:** Margaret Allard, Joshua Shubert, Muyinatu A. Lediju Bell

**Affiliations:** a Smith College, Department of Physics, Northampton, Massachusetts, United States; b Johns Hopkins University, Department of Electrical and Computer Engineering, Baltimore, Maryland, United States; c Johns Hopkins University, Department of Biomedical Engineering, Baltimore, Maryland, United States

**Keywords:** da Vinci^®^ robot, minimally invasive surgery, photoacoustic-guided surgery, robotic hysterectomies, surgical navigation, ureter, uterine arteries

## Abstract

Hysterectomies (i.e., surgical removal of the uterus) are the prevailing solution to treat medical conditions such as uterine cancer, endometriosis, and uterine prolapse. One complication of hysterectomies is accidental injury to the ureters located within millimeters of the uterine arteries that are severed and cauterized to hinder blood flow and enable full uterus removal. This work explores the feasibility of using photoacoustic imaging to visualize the uterine arteries (and potentially the ureter) when this imaging method is uniquely combined with a da Vinci^®^ surgical robot that enables teleoperated hysterectomies. We developed a specialized light delivery system to surround a da Vinci^®^ curved scissor tool, and an ultrasound probe was placed externally, representing a transvaginal approach, to receive the acoustic signals. Photoacoustic images were acquired while sweeping the tool across our custom 3-D uterine vessel model covered in *ex vivo* bovine tissue that was placed between the 3-D model and the fiber, as well as between the ultrasound probe and the 3-D model. Four tool orientations were explored, and the robot kinematics were used to provide tool position and orientation information simultaneously with each photoacoustic image acquisition. The optimal tool orientation produced images with contrast >10  dB and background signal-to-noise ratios (SNRs) >1.7, indicating minimal acoustic clutter from the tool tip. We achieved similar contrast and SNR measurements with four unique wrist orientations explored with the scissor tool in open and closed configurations. Results indicate that photoacoustic imaging is a promising approach to enable visualization of the uterine arteries to guide hysterectomies (and other gynecological surgeries). These results are additionally applicable to other da Vinci^®^ surgeries and other surgical instruments with similar tip geometry.

## 1 Introduction

Approximately 600,000 hysterectomies (i.e., surgical removal of the uterus) are performed each year in the United States, and approximately one in three women over age 60 will undergo this procedure in her lifetime.^1^ Hysterectomies typically follow the onset of medical conditions, such as endometriosis (where cells that are supposed to grow inside the uterus grow outside of it), uterine prolapse (where the uterus starts collapsing into the vagina), and uterine cancer. While hysterectomies may be performed laparoscopically, vaginally, or abdominally (i.e., open), hysterectomies are trending toward performance with robotic assistance, particularly with the da Vinci^®^ teleoperated surgical robot, due to increased dexterity, decreased hospital stays, three-dimensional stereoscopic visualization, minimal blood loss, and generally shorter recovery periods.^2^^–^^4^ While the da Vinci^®^ robot is used for other types of minimally invasive surgeries, such as radical prostatectomy,^5^ cardiac surgery,^6^^,^^7^ thyroid surgery,^8^ and thoracic surgery,^9^ one of the most common uses of the da Vinci^®^ robot is performing minimally invasive hysterectomies using small abdominal incisions to insert surgical tools.

**Fig. 1:**
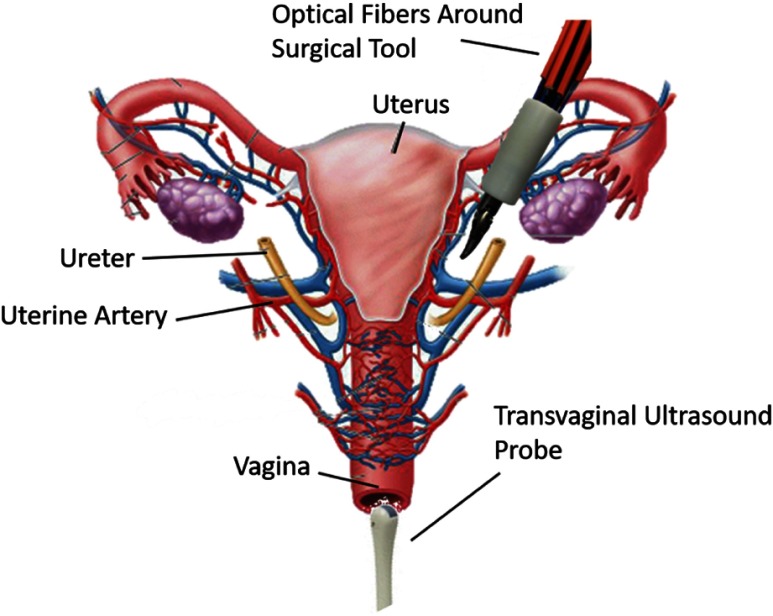
Proposed photoacoustic method for real-time imaging of the ureters and uterine arteries.

Once inside the body, to remove the uterus, the surgeon must cut and cauterize the uterine arteries while avoiding the ureter, the tube from the kidneys to the bladder, which is located a few millimeters from the uterine artery and crosses the uterine artery in some locations,^10^ as illustrated in Fig. 1. Ideally, accidental injuries to the ureter would be avoided and if they occur, recognizing them during surgery would be better than detecting them afterward. However, ∼50% to 70% of ureter injuries are undetected during surgery,^11^ which results in extensive, unplanned repeat surgeries and a more severe medical prognosis. For example, the longer the injury is undetected after the surgery, the more likely the development of urinoma (an encapsulated collection of urine near the kidneys) or complete kidney failure. This prolonged detection occurs because ureter injuries tend to be associated with few or no symptoms.^12^

Currently, the primary information available for ureter avoidance during robotic hysterectomies is the surgeon’s knowledge of general patient anatomy, the surgeon’s experience, and the surface view provided by the endoscope. This combination of information provides the surgeon with a general idea about the location of the ureter and the nearby uterine artery. However, intraoperative visualization of the ureter and uterine artery embedded in surrounding tissue remains as a challenge when surgeons are inexperienced or when anatomy is distorted due to endometriosis, fibroids, or very large uteri.^13^ Excessive electrocoagulation of uterine vessels also contributes to ureter injuries,^13^ indicating that a method to visualize vessels with more certainty prior to cauterization would be helpful.

Outstanding clinical challenges with ureter and uterine artery visualization during hysterectomies can potentially be addressed with the assistance of real-time image guidance. While ultrasound imaging is a potential option, it would be difficult to constantly maneuver an ultrasound probe to find the arteries and the ureter during surgery. In addition, the ureter and the uterine arteries are both hypoechoic targets and are therefore difficult to distinguish from each other in a real-time ultrasound image obtained with a drop-in probe. As an alternative, intraoperative near-infrared (NIR) fluorescence imaging has been proposed to offer real-time visualization of the ureter during surgery.^14^^,^^15^ This technique is based on the use of exogenous NIR fluorescent contrast agents that absorb light at one wavelength and emit light at a longer wavelength. Although this technique was successfully demonstrated in humans,^16^ the tissue penetration of NIR fluorescent light is limited to a few millimeters, thus the ureter must be relatively close to the surgical surface for it to be detected with this approach.^16^^–^^18^ In addition, this fluorescence imaging approach does not enhance visualization of the uterine arteries.

We propose to use photoacoustic imaging^19^^–^^21^ to visualize both the ureter and uterine artery during hysterectomies, with images that can be obtained as deep as several centimeters. This type of interventional or intraoperative photoacoustic imaging has previously been proposed to guide other surgeries, such as neurosurgery,^22^ fetal surgery,^23^ liver resection,^24^ and nerve-sparing radical prostatectomies.^25^ To guide teleoperated hysterectomies, we envision that optical fibers surrounding a da Vinci^®^ surgical tool would illuminate the surgical site. The uterine arteries, which have higher optical absorption than surrounding tissue, would absorb this light, undergo thermal expansion, and generate a sound wave to be detected with a transvaginal ultrasound probe, as illustrated in Fig. 1. Because urine has a low optical absorption,^26^^–^^28^ our overall vision includes contrast agents for ureter visualization. If a biocompatible contrast agent that is only sensitive to a narrow band of wavelengths^29^^–^^31^ is inserted into the urinary tract, the ureters can also be visualized with photoacoustic imaging, when the wavelength of the laser is tuned to the optimal wavelength of the contrast agent. With this approach, the surgeon can potentially have more information about the relative positions of the ureter and the uterine arteries. These photoacoustic images can be displayed on the same master console that the surgeon is using for teleoperation as previously proposed^32^ and demonstrated.^33^ In addition, because metal has a high optical absorption coefficient, the da Vinci^®^ tool can also be visualized in the photoacoustic image if it is located within the image plane.

The primary contributions of this paper are aimed at demonstrating the feasibility of photoacoustic-guided teleoperated hysterectomies in two stages. First, we design and prototype a custom light delivery system that surrounds a da Vinci^®^ scissor tool and demonstrate that this new combination of a da Vinci^®^ tool and custom light delivery system can be teleoperated successfully. Second, we investigate the optimal wrist orientations of the da Vinci^®^ scissor tool when our light delivery system is attached to the tool. These investigations were explored with a 3-D printed model of the uterine artery surrounded by *ex vivo* bovine tissue to provide the optical and acoustic scattering that would be caused by surrounding tissue in a hysterectomy procedure.

## 2 Methods and Materials

### 2.1 Experimental Setup

**Fig. 2:**
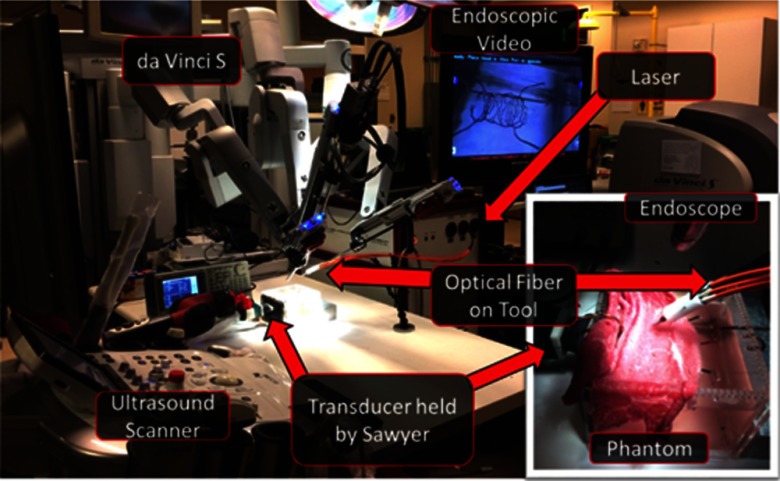
Photograph of the experimental setup. The inset shows a close-up view of the phantom used for our experiments, and it demonstrates the relative position of the ultrasound transducer and the optical fibers with respect to the vessel phantom that is covered by *ex vivo* bovine tissue. A close-up view of the uncovered phantom is displayed in the endoscopic video with the ultrasound transducer shown in the same orientation as that in the inset.

Our experiments were performed in a mock operating room that contained a da Vinci^®^ S robot, consisting of a master console (shown on the right side of Fig. 2), patient side manipulators (shown on the left side of Fig. 2), which are teleoperated from the master console, and an endoscope to visualize the surgical field (shown in the inset of Fig. 2). Only one of the patient side manipulators was used for our experiments, although three of these robot arms are shown in Fig. 2.

Our photoacoustic imaging system was positioned next to the mock operating table, which contained our experimental phantom. The photoacoustic imaging system was composed of an Alpinion ECUBE 12R ultrasound system connected to an Alpinion L3-8 linear transducer and a Phocus Mobile laser with a 1-to-7 fiber splitter^34^ attached to the 1064-nm output port of the laser. Ideally, the transmitted wavelength would be based on the optimal wavelength required to visualize structures of interest (e.g., 780 nm for deoxygenated hemoglobin). However, because we are imaging a black resin that is expected to have uniform absorption at all wavelengths, we identified 1064 nm to be suitable. The seven output fibers of the light delivery system surrounded a da Vinci^®^ curved scissor tool, and they were held in place with our custom designed, 3-D printed fiber holder. The da Vinci^®^ scissor tool was held by one of the patient side manipulators of the da Vinci^®^ S robot.

Our custom modular phantom (used in previous work^35^) was built from laser-cut acrylic pieces (held in place with silicone glue) and 3-D printed components. To simulate the uterine arteries, a 3-D model of the arteries around the uterus was designed and 3-D printed with black resin. This model was suspended by string through the holes of the phantom, and it is shown in Fig. 2, on the monitor displaying the endoscopic camera video feed.

The phantom was filled with water to permit acoustic wave propagation. The ultrasound transducer was fixed against the acoustic window of the phantom and held by a Sawyer robot (Rethink Robotics), which was used as a stable passive arm for the experiments to ensure that all images were acquired in the same image plane. A 1.5-mm thick layer of *ex vivo* bovine tissue was draped over the phantom (as shown in the inset of Fig. 2), to reside between the optical fiber and the vessels, and another layer of this same tissue was placed inside the phantom, between the 3-D model and the transducer. These tissues were placed to introduce both optical and acoustic scattering for photoacoustic imaging. Regarding the tissue placed to introduce optical scattering, a 1.5-mm thick tissue layer is realistic, based on our observations of robotic hysterectomy procedures. In addition, in our previous paper (which used the same setup^35^), we varied the tissue thickness from 1.5 to 4.5 mm and achieved sufficiently good images with thicker tissue layers.

### 2.2 Exploring Variations in Tool Orientations

The surgeon uses a wide range of surgical tools during the hysterectomy procedure. One required tool to sever the uterine artery is the curved scissor tool. This and other tools are used with dexterity that is similar to human hands. The wrist of the tool can therefore have multiple orientations during surgery, which could potentially impact the quality of photoacoustic images if a significant portion of the light is blocked by the tool.

**Fig. 3:**
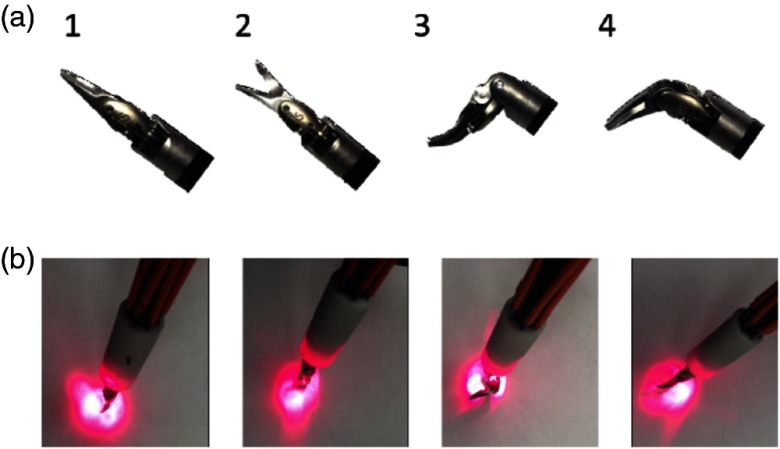
(a) Photographs of tool Orientations 1 through 4, from left to right, respectively. The trajectories of the scissor tool when placed in each of these four orientations are shown in Fig. 4. (b) Corresponding light profiles for tool Orientations 1 through 4, from left to right, respectively, acquired with 635-nm wavelength laser interfaced with the 1-to-7 fiber splitter. Note that although a 635-nm wavelength of light is shown to aid in the light profile visualization, a 1064-nm wavelength was used for the experiments presented in this paper, as noted in Sec. 2.1.

We explored variations in photoacoustic imaging with the four wrist orientations of the curved scissor tool shown in Fig. 3(a). Orientation 1 shows a straight tool (i.e., no bending of the wrist) with the scissors closed. Orientation 2 is the same as Orientation 1, but the scissors are open. The wrist is bent in Orientation 3 and the joint connecting the scissors is also bent. In Orientation 4, the wrist is not bent, but the joint connecting the scissors is bent. These orientations permitted passage of a varied amount of light, as shown in the photographs of Fig. 3(b), which were taken with a 635-nm laser light coupled to the input of the 1-to-7 fiber splitter surrounding the tool. Note that although a 635-nm wavelength was used to aid in the light profile visualization, a 1064-nm wavelength was used for the experiments presented in this paper, as noted in Sec. 2.1. When performing the experiments with 1064-nm wavelength pulsed laser light, the measured output energy per pulse for Orientations 1, 2, 3, and 4 was 1.44, 1.44, 1.40, and 1.36 mJ, respectively, and the average input energy was the same for all orientations.

### 2.3 Tracking Tool Positions and Orientations

The da Vinci^®^ robot arm with the custom light delivery system attached to the curved scissor tool was swept along the tissue surface, away from the portion of the 3-D model that was designed to mimic the portion of the uterine artery that crosses the ureter. This sweeping motion was proposed and demonstrated in previous work,^32^^,^^35^ and it would occur within the inflated abdominal cavity, above the uterus, within the same space that tools are manipulated in current robotic hysterectomy procedures. Because the phantom was covered in tissue, we used the photoacoustic image appearance of the uterine artery to determine the starting point of each sweep, and we stopped sweeping when the vessel was no longer visible in the photoacoustic image. Once we found the signal in a photoacoustic image, we used our knowledge of the underlying hidden vessel location to determine the direction of sweeping.

**Fig. 4:**
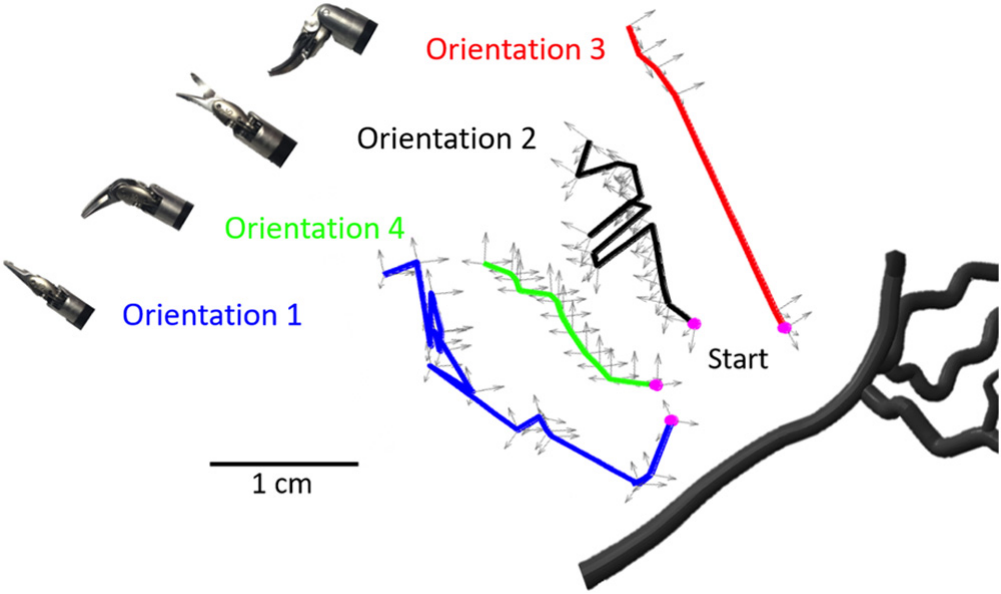
Trajectories of the scissor tool wrist with axes representing the wrist position and the da Vinci^®^ arm orientation. These trajectories are shown relative to the vessel branch that was imaged. The ultrasound probe was located at the bottom of this image. A video showing the sweeping motion and direction relative to the phantom is included as a separate file (see Fig. 5 for more details).

Photoacoustic images were acquired during each sweep for each tool orientation. The da Vinci^®^ robot kinematics were utilized to track the position and orientation of the tool, and we simultaneously acquired this tracking information with each image acquisition. The position of the coordinates corresponds to the position of the tool wrist and the orientation of the coordinates corresponds to the orientation of the da Vinci^®^ robot arm. These tracking coordinates are represented relative to the 3-D vessel solid model in Fig. 4, where each trajectory is mapped in a different color, and the pink dots represent the start of each sweep. The sweep for Orientations 1, 2, 3, and 4 contained 16, 19, 6, and 19 acquisition points, respectively. Orientation 3 had the fewest acquisition points because it was particularly difficult for the user to maintain this orientation during the sweep. With the exception of Orientation 1, the described sweeping motion was teleoperated from the master console of the da Vinci^®^ robot.

**Fig. 5:**
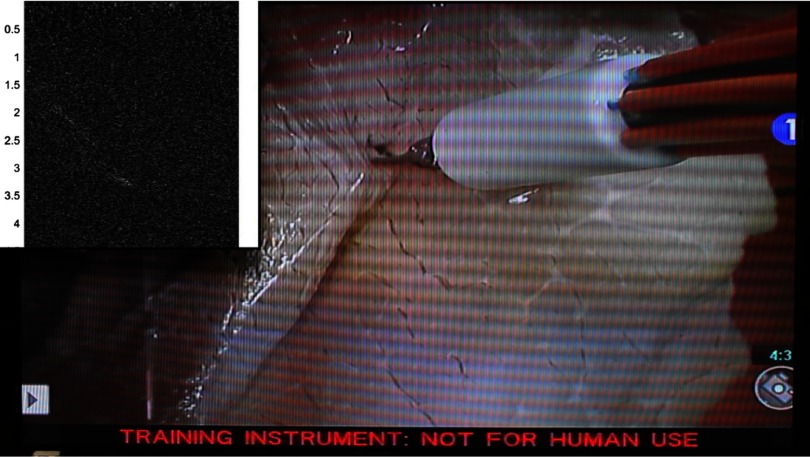
Still frame of video showing the sweeping motion of the da Vinci^®^ scissor tool, surrounded by our custom light delivery system. The inset in this video shows the simultaneously acquired photoacoustic images that correspond to the tool sweeping motion (Video 1, MPEG, 6.6 MB [URL: https://doi.org/10.1117/1.JMI.5.2.021213.1]).

A video demonstration of the teleoperated sweeping motion and the corresponding synchronized photoacoustic image acquisitions is uploaded as a separate file, with the first frame of this video shown in Fig. 5. The video shows fiber motions away from, then toward the location of the ultrasound probe, as viewed from the endoscope shown in Fig. 2. The inset shows corresponding photoacoustic images. This demonstration contains a small puncture in the tissue that was not present during the experiments. However, this puncture does not significantly affect the photoacoustic images, when compared to the point-like images of the vessels obtained when no tissue is present, as observed in our previous experiments without tissue.^35^ At the end of the video, we observe the scissor tool entering the puncture and clutter from the out-of-plane tool appears in the corresponding photoacoustic images.

### 2.4 Data Analysis

To determine if the amount of light blocked by the scissor tool affects our ability to see portions of the uterine artery, the percentage of this vessel visible in each photoacoustic image was measured and grouped by the associated tool orientation. These measurements were summarized with box plots, where the horizontal line inside each box displays the median value, and the upper and lower edges of each box represent the first and third quartiles of the dataset. The vertical lines connected to the boxes show the minimum and maximum values in each dataset, excluding outliers, which are shown as dots and defined as any value greater than 1.5 times the interquartile range.

Based on our prior knowledge that distance from a target can diminish target contrast,^22^^,^^32^ we additionally measured the contrast of each signal as a function of distance from the starting point for each orientation. Contrast was measured as follows:

$$\text{Contrast}=20\;\log_{10}(\frac{\mu_{\text{vessel}}}{\mu_{\text{background}}}),$$ (1)

where $\mu_{\text{vessel}}$ is the mean signal within a region of interest (ROI) inside the vessel signal (obtained by clicking along the vessel with a computer mouse and expanding the chosen point by ±20  pixels or ±0.4  mm, in the axial dimension) and $\mu_{\text{background}}$ is the mean of the signals in the background ROI, defined to be the same size and shape as the ROI, but translated by a distance of 0.6 mm to the right of the vessel. These measurements were performed using the beamformed radiofrequency (RF) data, and the ROIs were only defined once for each tool orientation.

**Fig. 6:**
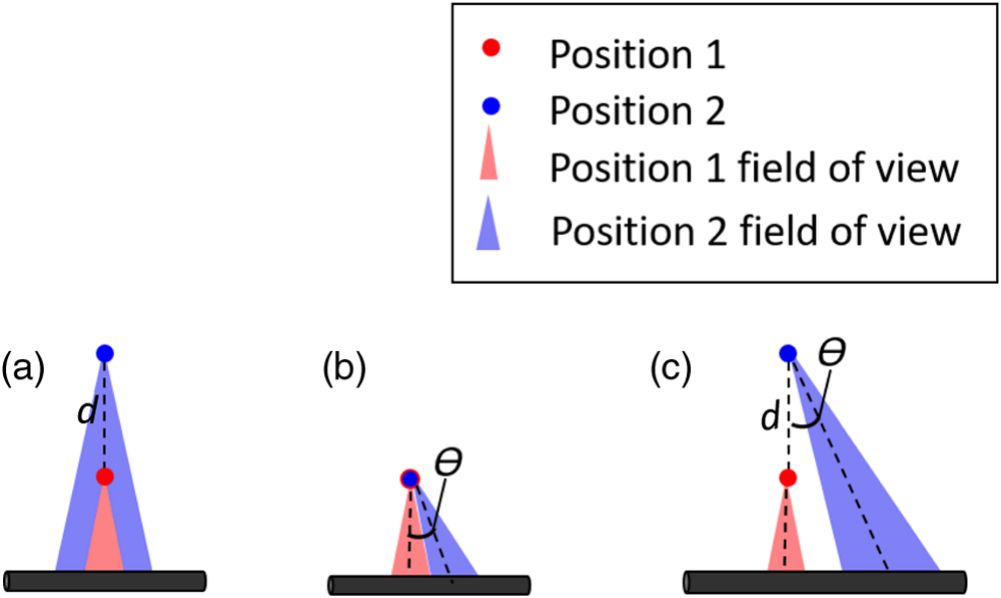
Effect of d and θ on vessel visibility. (a) When the distance d from a starting point changes and there is no change in θ, the original field-of-view remains the same. (b) When there is a change in θ and a small change in d, there is some overlap in the field-of-view. Larger changes in θ would cause less overlap. (c) When there is a large change in d and θ combined, there is a significant change in the field-of-view.

As illustrated in Fig. 6, the optical field-of-view of our light delivery system (which partially determines our ability to visualize a target) is affected by both distance and tool orientation. The light emitted from each of our seven optical fibers surrounding the tool has a conical shape, whose width is dependent on the numerical aperture of the fiber.^34^ Because of this conical light profile geometry, if we do not change our tool orientation and we move away from the target by a Euclidean distance, d, we have a greater chance of seeing the same target [Fig. 6(a)]. Similarly, we have greater tolerance to changes in the angle that defines the orientation of our tool in 3-D space, θ, if we are close to the target [Fig. 6(b)]. However, as shown in Fig. 6(c), the farther away we move from a target, the greater the impact variations in tool orientation will have on our ability to see the same target (or the same portion of a target). Thus, we define a new metric, dθ, to determine if the same portion of the vessel model was illuminated with each sweep. As it relates to our experiments, d is the Euclidean distance from the pink start point in Fig. 4, and θ is the angular difference between the orientation of the robot arm at the starting location (pink dot) and the orientation at any point along the trajectory.

Finally, based on our knowledge that the surgical tool tip will generate a photoacoustic signal and that these signals will manifest as clutter when the tool is not aligned with the ultrasound image plane, we measured the background signal-to-noise ratio (SNR) of each acquired photoacoustic image and grouped these images by tool orientation. SNR was measured as follows:

$$\mathrm{SNR}=\frac{\mu_{\text{background}}}{\sigma_{\text{background}}},$$ (2)

where $\sigma_{\text{background}}$ is the standard deviation of the signals within the ROI defined as all lateral positions spanning from 4 to 6 cm image depth (where clutter artifacts from the tool are most likely to appear), and $\mu_{\text{background}}$ is the mean of the background signals within this same ROI. These measurements were performed on the beamformed RF data. The fixed ROI is acceptable because the ultrasound probe was stationary throughout the experiments, and no vessel signals were observed below 4 cm in the corresponding ultrasound image.

## 3 Results

### 3.1 Vessel Visibility

**Fig. 7:**
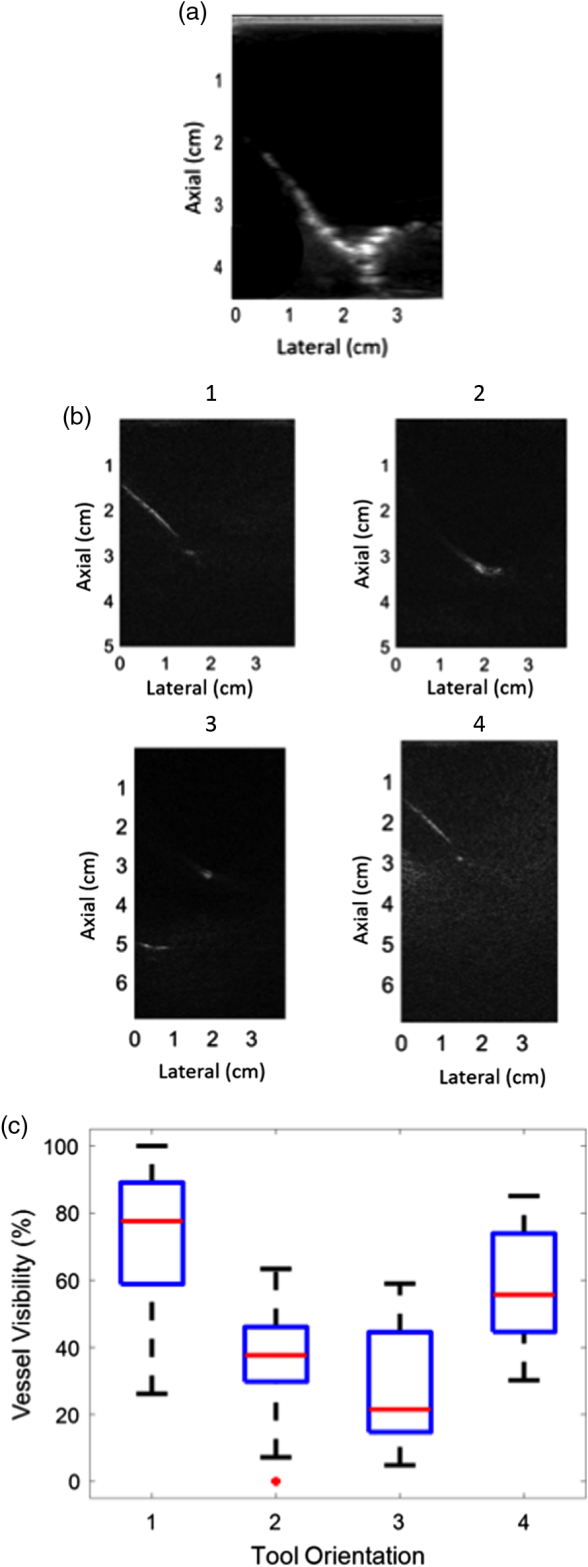
(a) Ultrasound image of the vessel phantom (acquired prior to the placement of tissue between the transducer and phantom in order to obtain a ground truth image for vessel visibility with minimal acoustic scattering). (b) Photoacoustic images acquired with tool Orientations 1 to 4 (indicated above each image). (c) Boxplot showing the percent of the vessel visible in each orientation.

An ultrasound image of the vessel branch visualized in our experiments is shown in Fig. 7(a). This ultrasound image was acquired with no tissue placed in front of the transducer (although tissue was present for the corresponding photoacoustic images). To summarize vessel visibility for each tool orientation, the length of the vessel as it appeared in each photoacoustic image was measured and normalized by the greatest length measurement overall. Note that the length of the vessel branch as it appears in the corresponding ultrasound image [i.e., Fig. 7(a)] is not considered in these length measurements. Instead, we considered the maximum length of the vessel visualized in photoacoustic images to represent 100% vessel visibility, which corresponded to the top left image in Fig. 7(b) (acquired with the scissor tool in Orientation 1). Figure 7(b) shows examples of photoacoustic images obtained with the remaining tool orientations (as indicated above each figure). The boxplot in Fig. 7(c) shows the distribution of vessel visibility percentages for each tool orientation. Orientation 1 had the greatest vessel visibility, which is intuitive because it blocks the least light. Orientation 3 had the lowest median of the four orientations, and it blocks the most light.

It initially appeared as if Orientation 1 achieved 100% vessel visibility because it permitted the passage of more light. While it may be generalized that the vessel visibility is related to the percentage of light that is blocked by the tool, this generalization is not consistent across all results. For example, Orientation 4 blocked more light than Orientation 2, but it produced images with greater vessel visibility, which indicates that there are additional factors to consider when characterizing vessel visibility.

### 3.2 Contrast and Distance Measurements

**Fig. 8:**
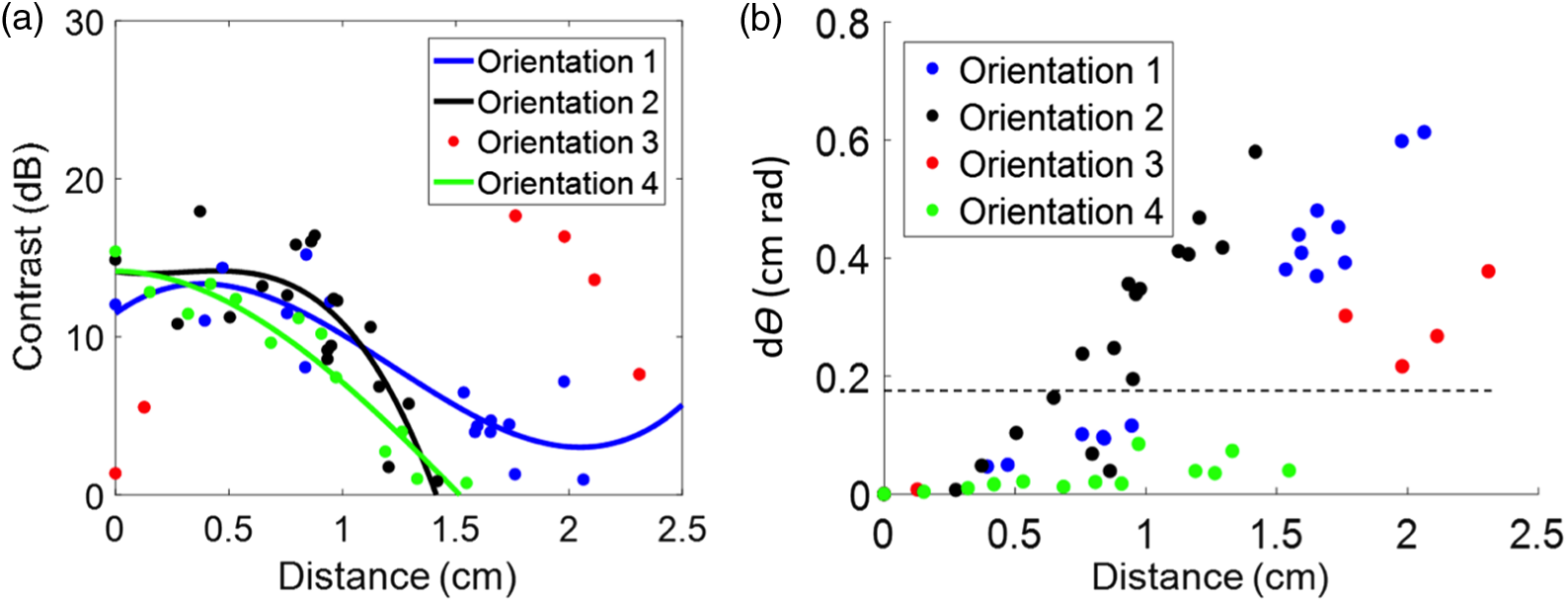
(a) Contrast measurements were plotted as a function of distance and fit using third-order polynomials. (b) The distance measurements were plotted as a function of our newly defined dθ term. A dotted horizontal line was added to show the separation between images acquired when the optical field-of-view covered the same region of the 3-D model as that of the initial starting point for each image (see Fig. 6 for more detail). This demarcation was visually confirmed for each photoacoustic image.

For each tool orientation, the measured contrast was plotted as a function of distance from the start of each sweep, as shown in Fig. 8(a). The four orientations were generally capable of producing high-contrast images (which is considered to be within the range of 10 to 20 dB). When images were acquired at a distance greater than 1 cm from the starting position, the contrast tended to drop below 5 dB, which is considered low contrast. The contrast measurements for each orientation were fit to third-order polynomials to demonstrate this trend, with Orientation 3 presenting an exception to these findings.

In order to understand what caused Orientation 3 to have a high contrast at 2 cm and a low contrast at 0 cm, both distance and angle were considered. Larger distances from the starting point are expected to be more affected by small angular changes, thus distance was plotted against our newly defined variable dθ in Fig. 8(b). We manually confirmed that images acquired with dθ>0.2 were never looking at the same part of the vessel as the image acquired at the beginning of each sweep. In the two low contrast images obtained with Orientation 3 (located near the starting position), the longer line of the vessel was visualized. In the higher contrast images obtained with Orientation 3 (located near 2 cm from the starting position), the bifurcation point of the vessel was visualized. This result indicates that even though the early images were close to the starting point, they were far from the long vessel branch (as shown in Fig. 4). Similarly, the images acquired with Orientation 3 farther from the start were viewing a portion of the vessel that was closer to the tool (also seen in Fig. 4, where the end trajectory of Orientation 3 is closer to the vessel bifurcation point than it is to the long vessel branch). This result confirms that dθ is an important metric to consider when characterizing vessel visibility with each sweep.

### 3.3 Image Clutter

**Fig. 9:**
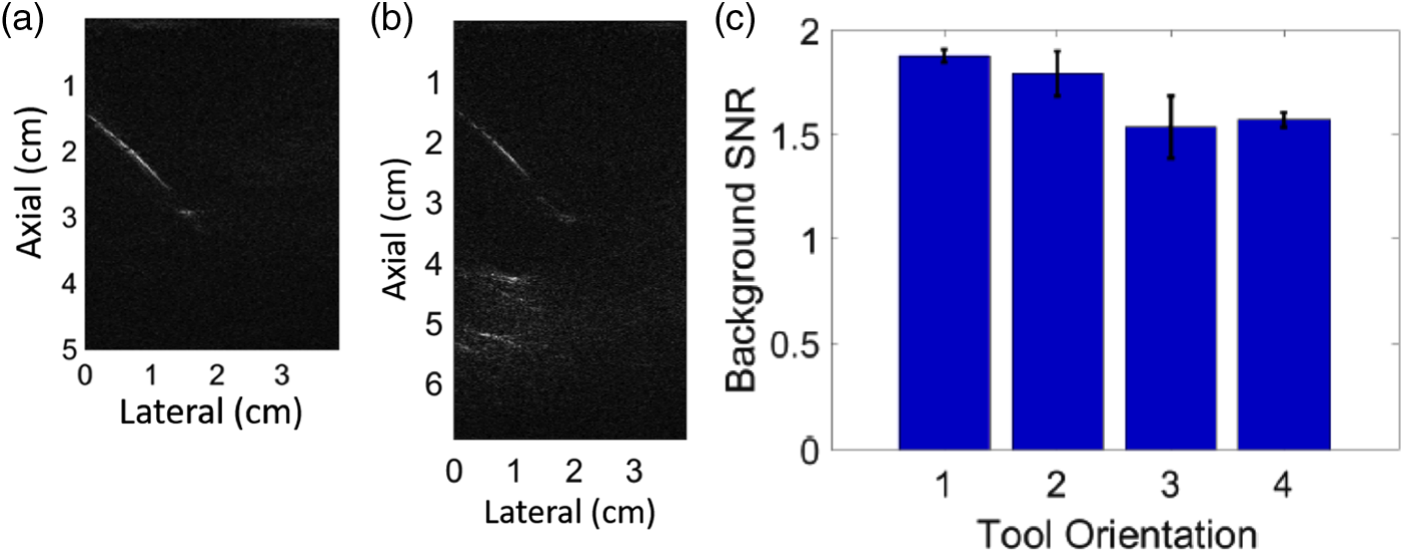
Photoacoustic images acquired with (a) Orientation 1, containing minimal acoustic clutter and (b) Orientation 4, containing acoustic clutter from the out-of-plane tool tip. The field-of-view was extended in the axial dimension of photoacoustic images from Orientation 4 in order for us to fully characterize and confirm the source of the extra signals. (c) Bar graph summarizing the mean ± one standard deviation of background SNR measured in all images from each tool orientation. Additional sample photoacoustic images from all orientations (including Orientations 2 and 3, which are not shown here) are shown in Fig. 7.

When light from the optical fiber is absorbed by the metal tip of the tool and this tool tip is outside of the image plane, the presence of acoustic clutter from the tool tip could complicate image interpretation. As each tool orientation absorbs varying degrees of light [based on the light profile images in Fig. 3(b)], tool orientation could impact the amount of acoustic clutter present in an image, particularly if the tool tip is outside of the image plane. Figure 9(a) shows an example of an image with minimal clutter acquired with the tool in Orientation 1, whereas Fig. 9(b) shows an image with more clutter from the tool tip, acquired with the tool in Orientation 4. The image depth in Fig. 9(b) was extended to fully capture and characterize the impact of the extra signals that appear deeper in the image. We know that these extra signals are caused by the tool tip based on three important observations. First, the tool tip was outside of the image plane. Second, the signals appeared with greater intensity when a significant portion of tool tip was in the path of the laser. Third, the real-time photoacoustic images showed that the location of these extra signals moved when the tool tip moved.

Background SNR was measured to quantify clutter in the image and plotted in Fig. 9(c). Images acquired with Orientation 1 had the least clutter and highest mean background SNR of 1.9. Orientation 2 produced images with slightly more clutter and a mean background SNR of 1.8, whereas Orientations 3 and 4 produced images with more clutter and mean background SNRs below 1.6. A lower background SNR indicates more clutter, and as expected, more clutter is present when the out-of-plane tool tip blocks a portion of the light.

## 4 Discussion

This work is the first to explore the feasibility of photoacoustic integration with the da Vinci^®^ surgical robot to potentially guide minimally invasive hysterectomies (and other gynecological surgeries that suffer from the same challenges with uterine artery and ureter visualization). We developed a specialized light delivery system to surround a da Vinci^®^ curved scissor tool to enable this investigation. We obtained photoacoustic images of our custom 3-D uterine artery model with reasonably high contrast within 1 cm from the artery [Fig. 8(a)]. To make use of the information provided by photoacoustic imaging, the spatial location of the surgical tool relative to the ultrasound imaging probe is required (unless the tool is located in the image plane and visible in the photoacoustic image). When the tool location is visible, it is sufficient to know the tool positions relative to other structures that are also present in the image without knowing the position of the tool relative to the ultrasound probe.^34^

In addition to developing a light delivery system and using it to obtain photoacoustic images, we defined a new term dθ, based on both distance from the target and relative orientation, that can be used to determine the likelihood that a surgeon will visualize the same structure while sweeping the tool. Generally, based on the results in Fig. 8(b), we can assume that surgeons will visualize the same region when dθ<0.2.

More generally, we found that the four orientations investigated were capable of producing high-contrast images (i.e., >10  dB). This high contrast was achieved for Orientation 3 at larger distances than 1 cm from the initial starting point when the relative angle was altered to obtain a dθ>0.2, which is indicative of a different vessel view for Orientation 3. In this orientation, we demonstrated that small angular differences (i.e., small θ) can alter the part of the vessel that is being visualized, particularly if the distance from the vessel is large. The implication of this finding for photoacoustic-guided surgeries with the da Vinci^®^ robot is that a surgeon who finds a low-contrast signal at any orientation and desires to improve this signal can consider locking all angular degrees of freedom prior to approaching the target to improve image contrast. Alternatively, the surgeon could consider switching to a coherence-based beamforming method that has been demonstrated to improve image contrast to guide surgeries,^35^ regardless of distance from the target^36^ (as long as the laser fluence is sufficient).

Acoustic clutter from out-of-plane tools could potentially be mistaken for the tool itself, causing confusion about the true tool location. Results indicate that images acquired with the tool in Orientation 1 produced the least clutter, while images acquired with the tool in Orientation 2 produced slightly more clutter. Orientations 3 and 4 produced images with the most acoustic clutter from the out-of-plane tool tip. This clutter could potentially be mitigated with advanced signal processing methods, including some recent advances in machine learning applied to photoacoustic beamforming.^37^^,^^38^ In addition, knowledge that the clutter appears deeper in the image could be used to ignore these clutter signals. However, these signals could also be mistaken for the tool residing in the image plane if they are not cleared from the image with advanced signal processing methods.

There are several factors that could determine the optimal tool orientation. Initially, it seemed likely that the optimal tool orientation was tied to the percentage of light that was blocked, which could be related to the percentage of a structure visible in the photoacoustic image, indicating that Orientation 1 is the most optimal orientation when creating photoacoustic images for surgical guidance with the da Vinci^®^ robot. This orientation is indeed optimal, but for two primary reasons that are not related to the percentage of the vessel visible. First, Orientation 1 produced high-contrast images when at least 1 cm away from the beginning of the sweep (which is also true of Orientations 2 and 4). Second, Orientation 1 produced the least acoustic clutter from the tool tip (Fig. 9). While Orientation 1 also provided the greatest percentage of vessel visibility (Fig. 7), this result seems to be more tied to dθ and the location of the tool relative to the vessel (rather than being tied to the percentage of blocked light). Additional work is required to determine the effect of tool orientation on vessel visibility when these other possible variables are held constant.

Although Orientation 1 is the optimal orientation as discussed above, we observe from Fig. 8(a) that all four orientations can produce helpful, high-contrast photoacoustic images. In fact, if a signal is found while in a different orientation, the surgeon is advised to lock θ before approaching in order to maintain the part of the vessel visualized in the image. This locking can be relaxed as the surgeon approaches the feature of interest and the contrast of the signal increases. Future work will explore automated methods to lock θ using contrast as a metric of distance from a desired target.

## 5 Conclusion

We demonstrated the feasibility of integrating photoacoustic imaging with the da Vinci^®^ robot in order to improve targeting of the uterine arteries during hysterectomies. Our integration included a specialized light delivery system to surround a da Vinci^®^ curved scissor tool. We additionally provide a detailed analysis of the optimal tool orientations for photoacoustic-guided surgeries using a scissor tool that partially blocks the transmitted light, indicating that the four orientations investigated have the potential to produce sufficient images for photoacoustic guidance. The optimal orientation involved no bending of both the tool’s wrist and the joint connecting the scissors. Thus, if a surgeon desires a clear photoacoustic image of the uterine artery or ureter with minimal confusion about the tool location, the best option is to straighten the tool’s wrist and close and straighten the scissors if possible. However, to avoid losing sight of a low-contrast signal, it is helpful to lock all angular degrees of freedom before approaching this signal of interest to improve its contrast (instead of adjusting the wrist to achieve the optimal tool orientation). Although the focus of this work is improving hysterectomies performed with a curved scissor tool attached to a da Vinci^®^ robot, our findings are applicable to other da Vinci^®^ tools, other types of da Vinci^®^ surgeries, and laparoscopic surgeries in general that may utilize instruments with similar tip geometry.
